# Evaluating a Web-based Training Curriculum for Disseminating Best Practices for the Care of Newborns with Neonatal Opioid Withdrawal Syndrome in a Rural Hospital, the NOWS-NM Program

**DOI:** 10.21203/rs.3.rs-2531394/v1

**Published:** 2023-02-16

**Authors:** Heather Pratt-Chavez, Heidi Rishel Brakey, Sarah G. Sanders, Juhee Patel, Tim Ozechowski, Chloe Stoffel, Andrew L. Sussman, Jessie Marquez, David R. Smith, Alberta S. Kong

**Affiliations:** University of New Mexico School of Medicine; University of New Mexico Clinical and Translational Science Center; University of New Mexico School of Medicine; University of New Mexico School of Medicine; University of New Mexico School of Medicine; University of New Mexico School of Medicine; UNM Comprehensive Cancer Center; Influents Innovations, Inc; Influents Innovations, Inc; University of New Mexico School of Medicine

**Keywords:** Neonatal opiate withdrawal syndrome, trauma-informed care, rural, provider, curriculum

## Abstract

**Background:**

The incidence of neonatal opiate withdrawal syndrome (NOWS) in the US has grown dramatically over the past two decades. Many rural hospitals not equipped to manage these patients transfer them to hospitals in bigger cities.

**Methods:**

We created a curriculum, the NOWS-NM Program, a mobile/web-based curriculum training in best practices. To evaluate the curriculum, we conducted pre- and post-surveys of NOWS knowledge, attitudes, and care practices, plus post-curriculum interviews and focus groups.

**Results:**

Fourteen participants completed both pre- and post-curriculum surveys. They indicated an increase in knowledge and care practices. A small number of respondents expressed negative attitudes about parents of infants with NOWS at pre-test, the training curriculum appeared to have no impact on such attitudes at post-test. Sixteen participants participated in focus groups or interviews. Qualitative data reinforced the positive quantitative results and contradicted the negative survey results, respondents reported that the program did reduce stigma and improve provider/staff interactions with patients.

**Conclusions:**

This curriculum demonstrated positive impacts on NOWS knowledge and care practices. Incorporating focus on core concepts of trauma-informed care and self-regulation in future iterations of the curriculum may strengthen the opportunity to change attitudes and address the needs expressed by participants and improve care of families and babies with NOWS.

**Significance:**

This project evaluates a novel curriculum covering best practices in care of infants with neonatal opiate withdrawal syndrome (NOWS) and is oriented toward supporting care in rural NM hospitals. We evaluated the curriculum with both quantitative and qualitative methods. Results support the effectiveness of the curriculum to increase competence of rural providers in the care of patients with NOWS. The NOWS-NM Program is a novel and effective mobile training tool, especially for under-resourced, rural hospitals.

## Background

The incidence of neonatal opioid withdrawal syndrome (NOWS) in the United States has grown in the last two decades from 1.5 out of 1000 births in 1999 to an estimated 20 out of 1000 births in 2016^1,2^. Infants and parents in rural and under-resourced areas are disproportionately affected^3^; from 2008 to 2017 New Mexico experienced at 573.9% increase in NOWS cases^4^. Rural hospitals face significant barriers to caring for infants with NOWS stemming from a need for training combined with under-resourced facilities^3,5^. Despite the increase in numbers of infants requiring treatment for NOWS, dissemination of best practices in care of this specific population has not kept pace, particularly in the specific challenges faced by hospitals in rural areas^6,7^.

Infants with NOWS are typically observed in an inpatient setting for at least 24–96 hours after birth and evaluated for clinical need of pharmacologic treatment^8,9^. First-line treatments for symptoms of NOWS are non-pharmacologic interventions^10,11^. Non-pharmacologic care includes creating low stimulation environments for infants, an emphasis on breastfeeding, and other methods that require guidance and support from medical staff for both the infant and the parent or care-giver^11,12^. Positive partnerships between families and medical providers are critical to the success of non-pharmacologic interventions.

Provider bias and judgement can erode positive collaboration between families of infants with NOWS^13,14^. Furthermore, for patients in rural settings, lack of training in best practice for infants with NOWS can lead to hospital transfer and separation of the family^3^. Accessible and evidence-based training in NOWS care for providers in rural areas can improve care of infants with NOWS^15^. Additionally, increasing provider comfort treating patients with NOWS may reduce hospital transfers.

The NOWS-NM Program curriculum was designed to empower and educate rural providers caring for patients with NOWS. A mixed methods research approach was utilized to evaluate the first version of the curriculum. Emphasis was placed on exploring barriers to care that rural providers face when treating families caring for infants with NOWS, and whether this intervention was successful for the providers who participated in the curriculum.

## Methods

We developed the NOWS-NM Program curriculum in response to requests and input from rural providers caring for infants with NOWS. The curriculum focuses on best practices and basic treatment for infants with NOWS and consists of 11 online modules. The topics include management of opiate use disorder during pregnancy, an introduction to evaluating the symptoms of NOWS with both the Finnegan and Eat, Sleep Console tools, non-pharmacologic care, and discharge planning.

One rurally-located hospital served as a pilot site for the evaluation of the curriculum where providers and staff at the hospital participated in surveys and interviews after viewing the modules. Additionally, a small group of providers in Alaska participated in the evaluation after watching 2 core modules of the curriculum to determine if it would be useful for their locations.

We used a mixed methods approach to evaluate the curriculum. To evaluate effectiveness using quantitative measures, viewers, such as nurses, physicians and advance practice providers, were asked to complete pre- and post-surveys regarding knowledge, attitudes, and practice. We adapted the survey from Romisher et al (2018)^14^ with permission. The survey, administered remotely using RedCap, had a 5-point ordinal response scale ranging from 1 = “strongly disagree” to 5 = “strongly agree.” We compared the results of the pre- and post-surveys by tabulating the percentage of participants responding “agree” or “strongly agree” to each item.

Subsequently, using a descriptive qualitative design, we invited the same participants to share their feedback in an interview or focus group. Interviews and focus groups were conducted June through October 2020 using Zoom videoconferencing software. All video was deleted immediately after the interaction. Audio files were deleted after they were transcribed for analysis. Some participants viewed the curriculum within the previous 8 weeks, however others viewed it one year prior. If it had been more than 8 weeks, participants were asked to review the curriculum before the interview or focus group. One person was unable to schedule an interview or focus group and provided feedback in writing. Interviews/focus groups lasted 20 to 60 minutes. We asked questions about their perspective and experience working with patients with NOWS; practice readiness; feedback about the NOWS curriculum; and changes made because of the curriculum. Using NVivo qualitative software^16^, one analyst coded all transcripts using an inductive, thematic, and iterative process. In this manuscript, we focus on themes related to changes in practice, knowledge, and attitude that occurred as a result of the curriculum.

The authors certify that this work was conducted in accord with prevailing ethical principles and reviewed by an Institutional Review Board.

## Results

### Quantitative Results

**Figure 1:**
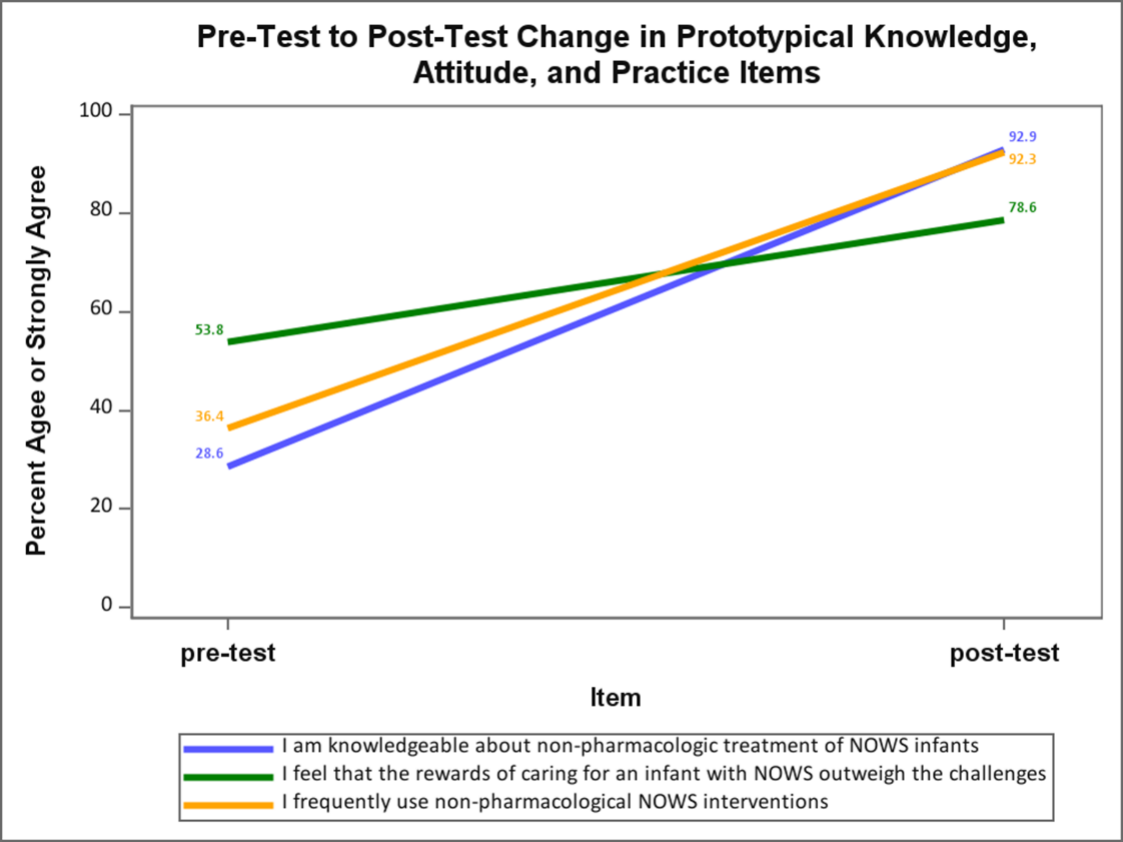
Pre-test to post-test change in prototypical knowledge, attitude and practice items

Pre- and post-surveys regarding knowledge, attitudes, and practice (KAP) of caring for infants with NOWS were completed by 30 participants: 23 from a rural New Mexico hospital; 7 from an interested hospital in Alaska. Of those, 14 participants completed both pre- and post-curriculum KAP surveys (2 MD, 11 RN and 1 NP). Survey results indicate substantial increases in knowledge of NOWS care across the 10 knowledge items: the average percent indicating “agree” or “strongly agree” increased from 31% at pre-test to 85% at post-test. Substantial increases were also reported on the 4 practice items: across the 4 practice items, the average percent indicating “agree” or “strongly agree” increased from 52% at pre-test to 91% at post-test (Fig. 1).

Changes on the 8 attitude items were less robust. On the 3 attitude items with positively worded stems (e.g., “I feel that the rewards of caring for an infant with NOWS outweigh the challenges.”), the percent indicating “agree” or “strongly agree” increased from 62–72%. On the 5 attitude items with negatively worded stems, only one item (“I believe that infants with NOWS should be cared for in a critical care environment.”) showed substantial improvement with 42% indicating “agree” or “strongly agree” at pre-test and 18% at post-test. None of the three items conveying negative attitudes regarding parents of infants with NOWS showed any improvement between pre-test and post-test. In fact, one item (“I find it frustrating when the mother of an infant with NOWS is unable to fully participate in infant care.”) showed deterioration with 25% indicating “agree” or “strongly agree” at pre-test and 42% responding with agreement at post-test.

In summary, substantial increases in NOWS knowledge and self-reported practice were obtained on the KAP survey. Most respondents agreed with the positively worded attitude items and pre-test and post-test. A few respondents expressed negative attitudes about parents of infants with NOWS at pre-test, the training curriculum appeared to have no impact on such attitudes at post-test.

### Qualitative Results

Sixteen providers participated in focus groups and interviews about the curriculum and their experience with NOWS (Table 1). Most were in NM (N = 11, 69%) and almost equally divided between registered nurses (N = 8, 50%) and providers (N = 7, 44%). Most participated in an interview (N = 9), while six participated in focus groups, and one submitted written feedback.

**Figure 2:**
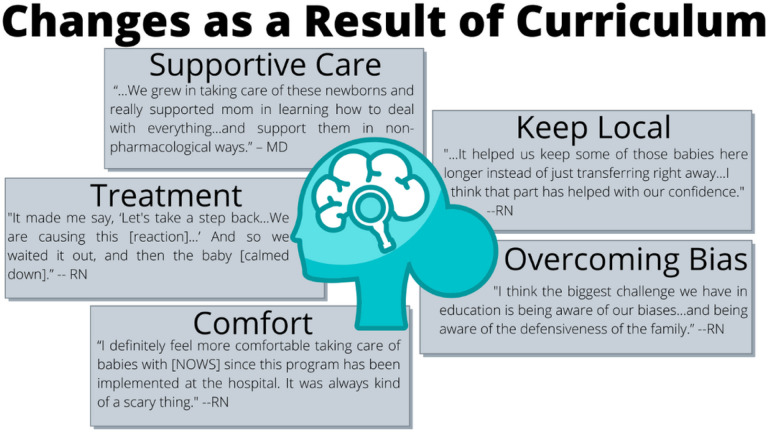
Summary of changes as a result of the curriculum

From those who discussed changes as a result of the curriculum, the following themes emerged: overcoming bias, feeling more comfortable and confident, changing knowledge and approaches to diagnosis and treatment, providing more supportive care, and keeping infants and families local (Fig. 2).

### Overcoming bias:

Some participants said the curriculum helped them or others view families dealing with opiate use disorder in a different light. Many referenced their own biases and the need to change their mindset and approach. One nurse summarized this change well:

…I felt like that was a huge part of our culture of nursing in our facility was that everyone is just like, ‘Oh, these druggies,’ and that’s what you initially think… I like how [the curriculum] was broken down…you need to remember that they all have a story, and that they’re all human, and that sometimes, the drugs take over their lives, but we’re here to help them and help their babies.

In addition, one provider said:

I think that treating parents as patients with a medical diagnosis has helped, rather than treating parents as criminals… so that’s the most important part about the entire curriculum is, I think if you change the hearts and minds… if you can help explain that probably the safety and the quality that the child deserves is to try to help them non-pharmacologically as well as you can and keep them, mom, families together and not fly them out, I think the changing of hearts and minds is the key.

### Feeling more comfortable and confident:

Nurses expressed feeling greater confidence and comfort working with infants with NOWS. One said:

I definitely feel more comfortable taking care of babies with neonatal opiate withdrawal syndrome since this program has been implemented at the hospital. It was always kind of a scary thing. And I felt like there wasn’t enough knowledge about what’s going on with them and what we need to expect and some of the rules and the guidelines and things. And I feel like things are more streamlined now, and it’s definitely more comfortable for everyone.

### Changes in knowledge and approaches to diagnosis and treatment:

Providers and nurses explained they learned how to better diagnose, score, and treat babies with NOWS. Babies are “scored” on their level of opioid withdrawal using standardized scales. One nurse said it helped the provider they worked with diagnose a baby with NOWS in a confusing situation, after re-watching the curriculum videos and reviewing their new policy. After diagnosis, participants said they use what they learned in the curriculum to score babies with NOWS. One nurse said, “I would refer to that and all my notes from the presentation. So, I felt like, clinically, that was the most useful thing for me that I used all the time.”

### Providing more supportive care:

Participants expressed their appreciation of the focus on consoling over scoring and have standardized their unit’s approach to scoring. One nurse explained how they used this knowledge in practice:

And it was just all of these stimulations we were causing to this baby, and then we got a high score. So, its kind of made me say, ‘Okay. Hold on. Let’s take a step back…We are causing this score, not that the baby’s scoring this way on its own. We did this.’ And so, we waited it out, and then the baby started scoring low.

### Keeping infants and families local:

Several nurses and providers emphasized the importance of keeping babies local, rather than sending them to a regional hospital, and this curriculum has helped them do that. One provider explained:

Before we started this program…every baby that was suspected of risk for withdrawal was just sent to [the big city]. So, it really wasn’t a thing, which was frustrating because I knew that we could deal with it. Nobody was comfortable doing that.

The results of the qualitative study contradicted the survey data regarding the effectiveness of the curriculum in addressing biases in providing care for patients with NOWS. Providers expressed more details and subtleties of their experience of change in the focus groups and interviews. The effect and interpretation of negative reports on the survey results are difficult to interpret in the light of the small sample size as well.

## Discussion And Conclusions

The NOWS-NM Program is a tool to increase access to care for an especially vulnerable population; infants with NOWS receiving care in a rural setting. Our work demonstrates that the curriculum increases competence and helps to address biases toward families dealing with substance use disorder. Reducing biases can ultimately reduce barriers for this population in accessing quality care in their home communities.

Previous work has demonstrated that neonatal intensive care (NICU) nurses often held negative judgement of parents with opiate use disorder and had a lack of competence in trauma-informed care^17^. This work demonstrates the effectiveness of the NOWS-NM Program curriculum in addressing this problem.

Our project also addresses the need for options for access, including mobile platforms, for continued training for providers caring for babies with NOWS and that training with current information regarding best practices increases quality care^15,18^. Our work also addresses the need to confront and correct biases that can lead to stigmatization of patients and negatively affect care^13,19^(Maguire et al., 2012; Recto et al., 2020).

The limitations of this project are related to working with small rural communities with small numbers of health professionals, leading to a smaller group of subjects. However, having a smaller group to pilot our curriculum was essential for refinement prior to future disseminations to other rural hospitals and teams.

## Figures and Tables

**Table 1:** Demographic summary of interview/focus group participants

	All Sites	NM	AK
**Participants** **Total N**	**16**	**11**	**5**
Registered Nurse, n (%)	8 (50%)	6 (55%)	
Provider (MD, NP), n (%)	7 (44%)	4 (36%)	
Licensed Social Worker, n (%)	1 (6%)	1 (9%)	
**Type of Feedback**			
Interview, n (%)	9 (56%)	9 (82%)	0
Focus Group, n (%)	6 (37%)	2 (18%)	4 (80%)
Written Feedback, n (%)	1 (6%)	0	1 (20%)

## Data Availability

The datasets used and/or analysed during the current study are available from the corresponding author on reasonable request.

## Abbreviations

AK: Alaska
KAP: knowledge, attitude and practice
NICU: Neonatal Intensive Care Unit
NM: New Mexico
NOWS: Neonatal Opioid Withdrawal Syndrome
NOWS-NM: the name of our NOWS curriculum
